# Consequences of children’s drowning: a systematic review 

**Published:** 2022-12

**Authors:** Fahimeh Barghi Shirazi, Shandiz Moslehi

**Affiliations:** ^*a*^ Department of Health in Disasters and Emergencies, School of Health Management and Information Sciences, Iran University of Medical Sciences, Tehran, Iran.; ^*b*^ Health Management and Economics Research Center, Health Management Research Institute, Iran University of Medical Sciences, Tehran, Iran.; ^*c*^ Department of Health in Disasters and Emergencies, School of Health Management and Information Sciences, Iran University of Medical Sciences, Tehran, Iran.

## Abstract

**Background:**

Among the emergencies, falling into the water and immersion is one of the common and at the same time preventable causes of injury and death in children. A heavy burden remains in less developed countries, which are less able to provide enough training. Thus, the present study aimed to identify the consequences of children’s drowning.

**Methods:**

In this systematic review, all studies published between 2000 and May 1, 2022, which were available in databases were reviewed including MEDLINE/PubMed, Scopus, Cochrane, Web of Science, ProQuest, Google Scholar, Iran Medex, Magiran, Scientific Information Database (SID), key journals, list of the references of the included studies, gray literature, and websites of relevant organizations. The findings were analyzed using thematic content analysis. The results were expressed according to PRISMA 2020 guidelines. The quality of the included studies was evaluated using relevant checklists.

**Results:**

The relevant databases were searched and 1966 studies were found. After removing duplicates, the titles and abstracts of 1230 articles were reviewed. A total of 32 articles presenting the consequences of children’s drowning were included in this systematic review. They were divided into 3 main categories; heart problems (heart failure), pulmonary effects (acute pulmonary edema, respiratory failure), and central nervous system damage (cerebral encephalopathy).

**Conclusions:**

The duration of immersion is a critical factor in the prognosis of drowning consequences. For this reason, teaching swimming lessons to children, particularly children over 4 years of age, proper use of personal flotation devices, continuous and reliable adult supervision, and pool fencing are currently the most effective strategies for reducing the consequences of drowning in the mitigation phase.

**Keywords:**

Consequence, Drowning, Children, Education, Emergencies

